# Thinking about thinking: implications of the introspective error for default-interventionist type models of dual processes

**DOI:** 10.3389/fpsyg.2014.00864

**Published:** 2014-08-07

**Authors:** Laura F. Mega, Kirsten G. Volz

**Affiliations:** Neural Basis of Intuition, Werner Reichardt Centre for Integrative Neuroscience, University TübingenTübingen, Germany

**Keywords:** dual-process theory, default-interventionist model, introspection, meta-consciousness

Imagine sitting in a grand hall, listening to the keynote lecture of the conference you are attending. At some point your thoughts drift-off. When you look around you, half of the audience is staring intently at their smartphones. You ask yourself: what is it about this talk that makes you unable to stay focused? Do you find any aspect enjoyable? How would you behave, if it were you standing at the podium? Answering questions such as these, you are engaging in a process known as introspection.

Introspection describes the ability to explicitly characterize experience. It enables one to say: “I am thinking about what I am thinking about.” In other words, introspection allows one to become meta-aware, that is, to have awareness of what one believes to be experiencing. Although agreement exists as to the fact that we all have and make experiences and therefore subjective experience seems indisputable (Schooler and Schreiber, 2004; Winkielman and Schooler, 2011), empirically gaining access to and knowledge of this subjective experience poses a great challenge. It requires us to put a subjective, internal experience into words, such as in the above-mentioned example. This raises the question, if the words we come up with are true descriptions of our experience, or confabulations. Specifically, the dissociation between experiential consciousness (the contents of experience) and meta-consciousness (the belief about the contents of experience) makes us fallible in appraising our own experiences. In some cases, this fallibility has been demonstrated to manifest in translational dissociations, that is, the distortion of experience in an attempt to recount or characterize it; this was termed the “introspective error” (Schooler, 2002). Even though—or perhaps because—the paradox of introspection has been studied extensively for a number of decades, it is almost paradoxical itself to find that the resulting implications for the ongoing debate about (dual-) process types in judgment and decision-making (JDM) and specifically for the most widely accepted and experimentally investigated *default-interventionist model* (D-I-Model), have not been considered thus far. In the present contribution we set out to fill this gap and point out the implications of the introspective error for the conceptualization of the D-I-Model.

In the (neuro-) scientific community, dual-process models of intuitive and deliberate JDM currently constitute the preferred theoretical construct (e.g., Lieberman et al., 2002; Strack and Deutsch, 2004; Glöckner and Witteman, 2010; Kahneman, 2011; Evans and Stanovich, 2013). These models have been built on the assumption that judgments are formed via two qualitatively distinct process types: automatic “intuition” and controlled “deliberation” or “reflection.” In recent years, an immense influx of publications has arisen, either fervently defending or criticizing the dualistic distinction between rapid, autonomous, intuitive processes and slower, thoughtful, reasoning processes of higher order. In their most recent publication on dual-process models, Evans and Stanovich (2013)—henceforth referred to as E&S—described their concept as one, which assumes that automatic processes (Type 1, T1) yield default responses unless an intervention by higher order reasoning processes (Type 2, T2) is needed; a model, which has been termed the D-I-Model. We will focus on this current-most description of dual-process theory, since it constitutes the predominant model being intensively discussed by leading authorities in the field^1^. E&S split the attributes of both process types into defining (necessary/sufficient) and correlated features. The defining features listed for T2 processing are working memory capacity and cognitive decoupling. These are seen to be central in order to be able to reason hypothetically and distinguish supposition from belief, thereby aiding “rational choices by running thought experiments” (E&S, p. 236). Importantly, cognitive decoupling requires a re-representation of automatic (T1) processes so as to be able to interfere with their output. In that way T2 processing allows for “metarepresentational and simulation abilities,” and is thus a form of meta-consciousness. T1 processing, in contrast, is defined as encompassing both “innately specified processing modules or procedures and experiential associations that have been learned to the point of automaticity” (E&S, p. 236). Explicitly opposing a general “good-bad thinking idea,” the D-I-Model assumes T1 processes to lead to correct answers in benign environments, i.e., whenever the decision maker can use overpracticed cues. However, as soon as conditions for successful T1 processing are not fulfilled (e.g., novel situations), T2 processing will have to intervene on the default intuition. E&S argue that due to peoples' limited capacity of central cognitive resources, T1 processes inevitably will be relied on in most situations. The disposition to override the default intuition and to replace it by effective T2 reflective reasoning is suggested to be a function of several factors; an important one being “measurable thinking dispositions that are inclined toward rational thinking and disinclined to accept intuitions without checking them out” (p. 237)^2^. In other words, cognitive decoupling allowing a re-representation of automatic T1 processes seems to be decisive for intervention processes to become effective.

Literature on the introspective error, however, poses a challenge for this dual-process view insofar as it has been shown that re-representing subjective experience can lead to biases and incorrect decisions. Notably, this counterintuitive finding is not limited to T1-specific situations, where overlearned cues elicit the right answer, but also occurs in situations where the problem is hard to solve directly from previous experience or from previously stored cue validities. We will outline how the empirical results on introspection and meta-consciousness, presented by Schooler and others, are incongruent with the D-I-type models' assumption of reflective processes coming to the rescue of automatic response and will sketch a default-disruptive option. Therein, analytical introspection does not come to the rescue of intuitive, holistic recognition but rather disrupts this process, leading to changes in preference and even creating false outcomes (e.g., erroneous memories).

## Verbal overshadowing: an exemplary case

The verbal-overshadowing effect, first described by Schooler and Engstler-Schooler (1990), reveals a source of error in verbally describing a non-verbal stimulus: When individuals verbally introspect (i.e., attempt to describe in great detail) about complex non-verbal stimuli (e.g., recognizing a previously seen face, or the reasons for choice preferences), disruption can ensue. Particularly, individuals show markedly worse performance and make less optimal choices when asked to verbally introspect. In the words of Schooler and Schreiber (2004): “Verbal introspection fails to adequately capture ineffable experience, breaking them apart in a manner that makes it difficult to put back together” (p. 24). Interfering effects of verbalization have, for example, been found in a task requiring participants to watch a short video of a bank robbery and later attempt to identify the robber from a photo array. Those participants who had previously written a detailed description of the robber's appearance were markedly worse on the identification task than the control group (Schooler and Engstler-Schooler, 1990). The engagement of meta-conscious representation of subjective experience for subsequent production of a verbal description from memory actually led to a distortion of witness' memory, producing false outcomes. Schooler and colleagues posit that dissociations and omissions such as these can occur even when participants simply think aloud—concurrently or retrospectively—to the ongoing experiment. The authors reason that these distortions are due to the fact that participants are forced to verbally re-represent inherently non-verbal experiences (Schooler et al., 1993; Lane and Schooler, 2004; Winkielman and Schooler, 2011). This argument points to the *introspective error* mentioned above, wherein meta-consciousness is seen to misrepresent or distort underlying experience. In other words, the reflective mind lacks awareness of its own subjective state. This, however, would on the other hand be required in order to monitor when an intervention is necessary, according for instance to the D-I-Model.

The verbal overshadowing effect is not limited to visual introspection. Similar evidence comes from studies on preferential choice. Wilson and Schooler (1991) compared college students' preferences for courses with the ratings of experts. Students who were asked to introspect, i.e., analyzed the reasons why they preferred some courses over others, or evaluated attributes of all courses, made choices that corresponded less with experts' opinions than the choices of control subjects.

The two main points in discord with D-I-type models of dual-processes that are raised by the verbal overshadowing effect are as follows:

1. Verbal description of non-verbal memories induces distorting reflective processes (Jack and Roepstorff, 2002).

According to D-I-type models, T2 reflective processes are called upon to intervene on default answers in situations beyond those relying on innate or conditioned response capacities. Here, the engagement of T2 processing is assumed to be more likely to find the (normatively) correct answer. However, evidence from the study of introspection shows that performance may be less accurate when reflective strategies are applied (Dunning and Stern, 1994). Thereby, intervention by reflection disrupts performance (e.g., face recognition performance) rather than enhancing it.

1. “Analytic introspective processes induced by describing memories can disrupt holistic non-verbal recognition processes” (Schooler and Schreiber, 2004, p. 25).

In the above-mentioned example of the eyewitnesses, as we understand it, the D-I-Model would predict the default rise of a gut reaction (T1) to identify the perpetrator. When overridden by careful reflection (T2), the correct person should be remembered. Instead, as mentioned above, the opposite is true. Importantly, this misrepresentation of underlying experience is not explained by a monitoring failure. The monitoring failure account describes the introduction of bias, not from a lack of appropriate knowledge or cognitive resources (“mindware”) but a failure to call on this knowledge when it would be needed (De Neys and Bonnefon, 2013). However, the distortion of underlying experience by recall and verbalization is qualitatively different from the failure to draw on the appropriate knowledge. In misrepresenting (subjective) experience, the knowledge of the occurrence of experience needs to be actively called upon by meta-consciousness but the belief about what has been experienced does not align with what was actually experienced. Taken together, these findings lead us to propose an opposing view to the D-I-Model to describe subjective, non-verbalizable experiences; this is the “default-disruptive view.”

## The default-disruptive view

We propose tasks requiring introspection about inherently non-verbalizable processes as examples in which a default-disruptive option might more closely represent a mapping of people's cognitive processes as opposed to the current D-I-type models.

Tasks requiring the re-representation of inherently subjective conscious experience (what could be seen as being processed by the “autonomous mind”^3^) may elicit a translational dissociation between experiential consciousness and meta-consciousness (“reflective mind”). The recruitment of T2 processes disrupts the default response in non-verbalizable experiences, leading to:

- Distortions of underlying experience (e.g., verbal overshadowing effect)
- Decline in performance (e.g., speed)
- Decline in accuracy (e.g., recognition)

The introspective error challenges D-I-type dual-process models precisely because T1 processes are by their definition affect-laden decisions, based on a gut feeling primarily reflecting (non-verbalizable) experience (Betsch, 2008). Thus, overriding intuitive responses and replacing them by T2, reflective reasoning stringently requires a re-representation of subjective experience—raising the issues addressed above.

The challenge of the introspective error is all the more important since we are constantly encouraged, by self-help books and the like, for example, to carefully re-think (important) decisions in order to make the right choice. For instance, we can vividly imagine a police officer encouraging an eyewitness to carefully reconsider her response, emphasizing the implication a false statement would have; ironically, in doing so the police officer will foster exactly this outcome. Thus, dealing with the implications of research on the introspective error is not only relevant for the conceptualization of D-I-type dual-process models, but additionally has considerable implications for real-life decision making.

In summation, the “default-disruptive view” is a preliminary approximation to an alternate account of dual-process models in situations requiring introspection on internal processes. However, E&S themselves state that they “view the development of dual-process theories as an evolving project. Just as [dual-process theories] have developed and changed a great deal in the past decade, we expect this process to continue.” (p. 237). In this vein, our work provides a starting point and a fresh view for this evolutionary process.

### Conflict of interest statement

The authors declare that the research was conducted in the absence of any commercial or financial relationships that could be construed as a potential conflict of interest.

## Footnotes

^1^Compare the recent debate in the journal “Perspectives on Psychological Science,” Volume 8, 2013.

^2^A feature of human intelligence, as E&S assert.

^3^In keeping with Stanovich's “Tripartite Model of the Mind.”
